# Acute disseminated melioidosis giving rise to pneumonia and renal abscesses complicated with thrombotic thrombocytopenic purpura in a post partum woman: a case report

**DOI:** 10.1186/s13104-017-2997-7

**Published:** 2017-11-29

**Authors:** Piyumi Sachindra Alwis Wijewickrama, Rohini Weerakoon

**Affiliations:** 1 Postgraduate Institute of Medicine, University of Colombo, Colombo, Sri Lanka; 2 Teaching Hospital Karapitiya, Galle, Sri Lanka

**Keywords:** Melioidosis, Pneumonia, Renal abscesses, Post partum, Sepsis, Thrombotic thrombocytopenic purpura

## Abstract

**Background:**

Melioidosis is an established endemic infection in Sri Lanka, caused by *Burkholderia pseudomallei*, a gram negative bacterium distributed in saprophytes in soil and surface water. Main mode of transmission is via percutaneous inoculation. Pneumonia is the most common presentation in acute disease.

**Case presentation:**

We report a 33 year old previously healthy Sinhalese female with an occupational exposure to surface water in paddy fields, who was on postpartum day 6 following an uncomplicated pregnancy and delivery via an elective caesarian section. She presented with a 1 day history of breathlessness, preceded by a brief episode of fever. She had occasional right side coarse crackles and pitting oedema of both lower limbs. Shortly after admission, she developed type one respiratory failure needing invasive mechanical ventilation. Initial chest x-ray revealed slight obliteration of right medial diaphragmatic border while echocardiogram revealed moderate pulmonary hypertension. Computed tomography pulmonary angiogram excluded a pulmonary embolism, but revealed bilateral multi-lobar consolidation. Abdominal computed tomography demonstrated bilateral pyelonephritis with renal abscesses. As initial cultures were inconclusive, melioidosis antibody levels were done due to high degree of suspicion, which was found to be positive with a titer of 1:2560. A diagnosis of melioidosis was made based on the suggestive clinical picture, exposure history and the highly positive antibody level. She developed left side focal seizures together with thrombocytopenia and microangiopathic haemolytic anemia, suggestive of thrombotic thrombocytopenic purpura. Magnetic resonance imaging of brain was negative for cerebral abscesses but revealed extensive minute haemorrhagic foci throughout the cerebrum. Thus, the final diagnosis was acute melioidosis causing pneumonia and renal abscesses, complicated with thrombotic thrombocytopenic purpura and sepsis. She demonstrated dramatic response to high dose meropenem and co-trimoxazole along with plasmapheresis. Four weeks after treatment, the antibody titer came down to 1:320. Melioidosis antibody was absent in the baby.

**Conclusions:**

This case was challenging as it was an atypical presentation of melioidosis during postpartum leading to a diagnostic conundrum. This highlights the need to look into the effect of pregnancy and postpartum as added risk factors. High index of suspicion is necessary to avoid diagnostic delays.

## Background

Melioidosis is currently an established endemic infection in Sri Lanka [1]. The causative organism is *Burkholderia pseudomallei*, a gram negative, oxidase positive bacillus distributed in soil and surface water [2]. This organism is known to be widely distributed in irrigated lands such as paddy fields. The main mode of transmission is via percutaneous inoculation following exposure to contaminated soil or water [3]. Sri Lanka is a tropical country where rice is the staple food and paddy cultivation is a major livelihood especially in rural areas. This could be a contributory factor for the increasing numbers of melioidosis cases reported in the country in the recent past.

The incubation period is 1–21 days and most are known to be asymptomatic [2]. In up to 80%, the disease occurs in the presence of a risk factor such as diabetes mellitus, chronic lung diseases, chronic renal diseases, malignancy, thalassemia, glucocorticoid therapy and heavy alcohol use. Therefore, this is regarded as an opportunistic infection which is unlikely to lead to severe, fulminant disease in previously healthy persons [4, 5]. The disease has two distinct clinical entities as acute and chronic. Out of these, acute disease predominates, with pneumonia being the most frequent mode of presentation. The affected can also have other organ involvement causing genitourinary infection, skin infection and abscesses, soft tissue abscesses, septic arthritis and encephalomyelitis [2, 6]. Chronic disease can masquerade as tuberculosis or malignancy, leading to pneumonia, recurrent skin and internal abscesses [2].

Definitive diagnosis requires the culture of oxidase positive, gentamycin and polymyxin resistant gram negative bacillus. Diagnosis is supported by the positivity of *B. pseudomallei* agglutinating antibody or polymerase chain reaction (PCR). Culture of gram negative bacillus without any positive confirmatory tests and rising or a single very high antibody titer in a clinical setting consistent with melioidosis are considered supportive for the diagnosis, but not definitive [7].

We report an atypical presentation of melioidosis in a previously healthy, young female during postpartum period with severe bronchopneumonia, pyelonephritis and renal abscesses, complicated with sepsis and thrombotic thrombocytopenic purpura, leading to a diagnostic conundrum.

## Case presentation

A 33 year old previously healthy Sri Lankan, Sinhalese female, from the southern part of the country, who was on the post partum day 6, following an uncomplicated pregnancy and delivery via an elective caesarian section, presented with acute breathlessness of Medical Research Council (MRC) dyspnoea scale grade 4, for 1 day duration, which was associated with a non productive cough for 3 days. She did not give a history of orthopnoea, paroxysmal nocturnal dyspnoea or chest pain. She had an episode of low grade fever on post partum day 1, which settled spontaneously within 24 h. There was no fever on the day of admission. The rest of systemic inquiry was unremarkable. She works as a clerk in the local divisional secretariat and her work involves field visits to the paddy fields and farms with exposure to surface water and mud. On examination, she was overweight with body mass index (BMI) of 28 kg/m^2^. She was afebrile, and was neither pale, nor icteric. She had bilateral symmetrical mild, pitting lower limb oedema without signs of inflammation. She was in respiratory distress with dyspnoea at rest and tachypnoea. There were occasional coarse crepitations in the right lower zone. Her pulse rate was 130 per minute and blood pressure was 100/70 mmHg on admission. Her jugular venous pressure was not elevated. She had no organomegaly and her neurological examination including fundi was normal.

**Fig. 1:**
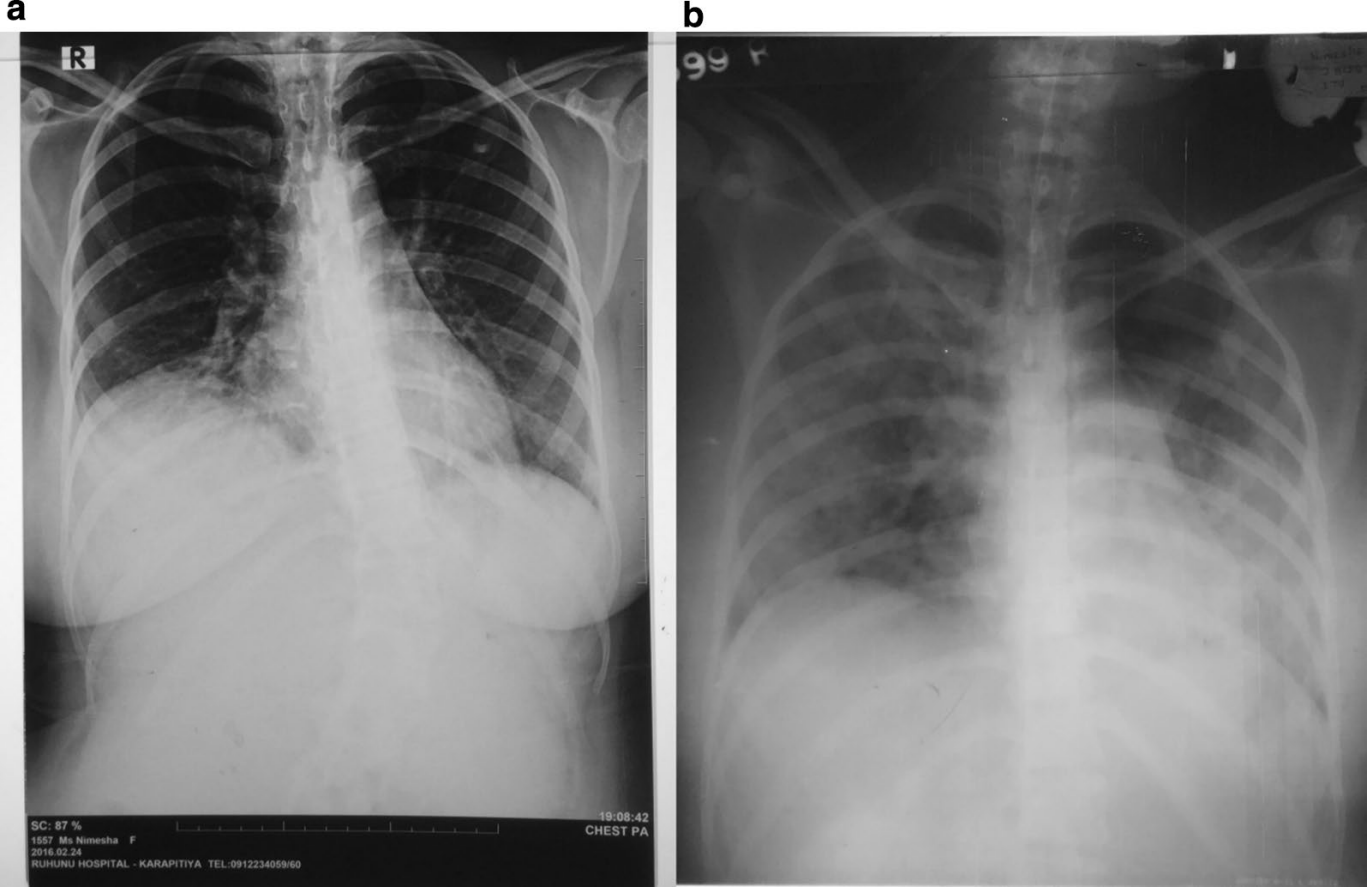
**a** Chest radiograph on admission. Shows slight obliteration of medial half of diaphragmatic border suggestive of right lower lobe consolidation. **b** Chest radiograph on day 3. Shows multi lobar consolidation

She developed type I respiratory failure within few hours of admission, needing invasive mechanical ventilation. Her chest x-ray on admission revealed slight obliteration of medial half of the right diaphragmatic border, suggestive of right lower lobe consolidation (Fig. 1a). Her electrocardiogram (ECG) showed sinus tachycardia and two dimensional echocardiogram revealed mild to moderate pulmonary hypertension with ejection fraction 60%. Haemoglobin was 10.2 g/dl, and she had a high total white blood cell (WBC) count of 11,000/µl with neutrophilic leukocytosis of 90.6%. She had thrombocytopenia since admission with a platelet count of 74,000/µl. Her C reactive protein (CRP) was 96 mg/l and Erythrocyte Sedimentation Rate (ESR) 100 mm in the 1st h. Her D-dimer level was slightly elevated at 4.8 mg/l (< 0.2 mg/l). The blood picture on admission showed normochromic normocytic red cells without fragmentation or polychromasia, mild neutrophilic leukocytosis with left shift and moderate thrombocytopenia, which was suggestive of sepsis. Her blood sugar was normal (80 mg/dl).

**Fig. 2:**
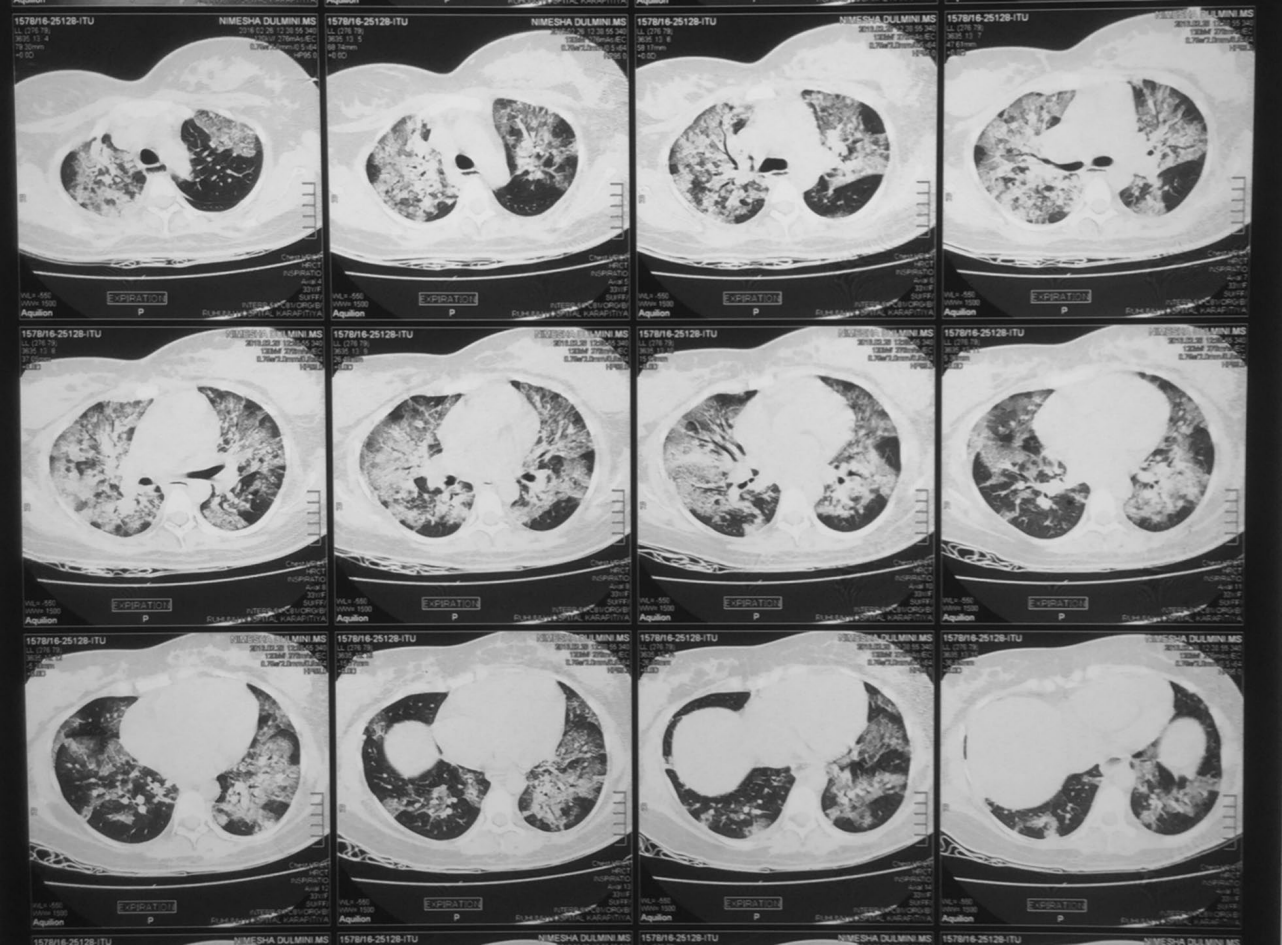
High resolution computed tomography of chest. Shows ground glass opacities throughout bilateral lung fields with multi lobar consolidation

Other initial investigations are summarized in Table 1. Based on these investigations, our initial differential diagnoses were severe pneumonia and acute pulmonary embolism. Her lower limb venous Doppler was negative for venous thrombi. Computed tomography pulmonary angiogram (CTPA) excluded pulmonary embolism. High resolution contrast tomography (HRCT) chest showed inhomogeneous, ground glass opacities throughout bilateral lung fields with consolidation in the right middle lobe, superior segment of left lower lobe and posterior segment of right upper lobe (Fig. 2).

**Table 1:** Initial investigation summary

Investigation	On admission	Day 6
Haemoglobin	10.2 g/dl	7.3 g/dl
WBC count	11.4 × 10^9^/l	23.8 × 10^9^/l
Neutrophils	90.6%	92%
Platelets	74,000/µl	47,000/µl
CRP	96 mg/l	185 mg/l
ESR	100 mm 1st h	
pH	7.31	
Partial pressure of carbon dioxide	28 mmHg	
Partial pressure of oxygen	68 mmHg	
HCO_3_^−^	14.1 mmol/l	
Lactate	5.4 mmol/l	
Urine full report	Pus cells—5–6 per high power field, red blood cells—25–30 per high power field, proteins—nil, Sugar—nil	
Serum creatinine	116 µmol/l	122 µmol/l
Serum sodium	134 mmol/l	144 mmol/l
Serum potassium	4 mmol/l	4.1 mmol/l
Aspartate aminotransferase	53 U/l	84 U/l
Alanine transaminase	50 U/l	52 U/l
International normalized ratio	1.0	1.1
Activated partial thromboplastin time	29 s	27 s

The patient was started on broad spectrum intravenous antibiotics (ceftriaxone 2 g 12 hourly and clarythromycin 500 mg 12 hourly). Despite antibiotics, she developed high grade fever from day 2 of admission. Chest radiograph on day 3 showed bilateral multi lobar consolidation (Fig. 1b). Abdominal ultrasonography revealed evidence of bilateral pyelonephritis with multiple abscesses in both kidneys.

While her sputum culture isolated a moderate growth of Coliform species, blood and urine cultures yielded negative results. Broncho-alveolar lavage fluid (BALF) culture done on day 5 was positive for *Stenotrophomonas maltophilia,* which was confirmed via Analytical profile index 20E. This is a gram negative bacterium known to colonize the respiratory tract in hospitalized or ventilated patients, rather than cause infection. BALF culture was negative for fungi and mycobacteria. PCR for *Mycobacterium tuberculosis* in BALF was also negative. Her retroviral screening, venereal disease research laboratory test, *Treponema pallidum* haemagglutination assay, Influenza A & B real time PCR assay, as well as autoimmune panel including Anti nuclear factor, Anti-neutrophil cytoplasmic antibody and Rheumatoid factor were negative.

**Fig. 3:**
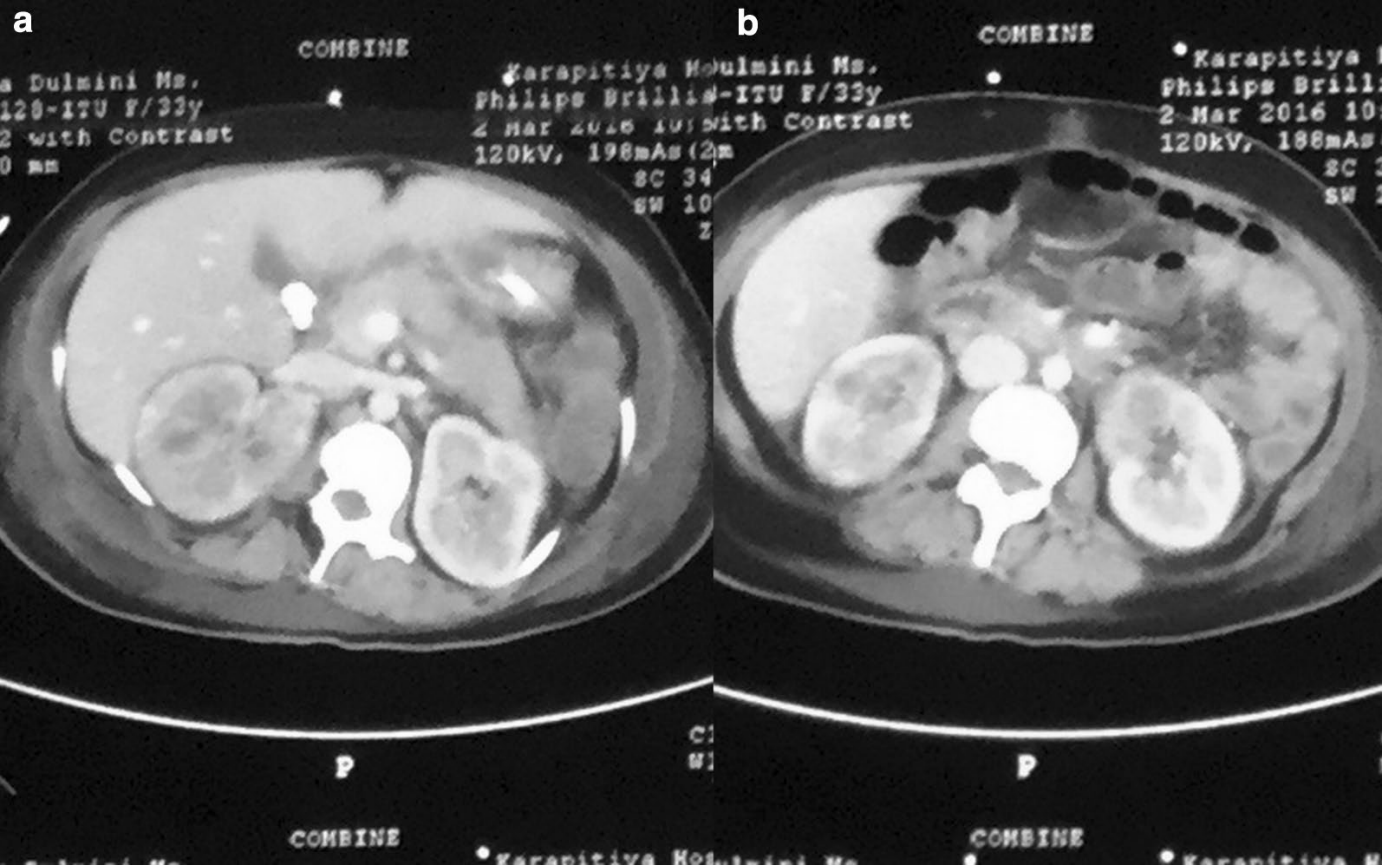
Contrast enhanced computed tomography of abdomen. Showing bilateral pyelonephritis with abscess on right upper pole (**a**) and early abscess formation on left lower pole (**b**)

Despite antibiotics, patient continued to have high fever and developed left side focal seizures on the sixth day after admission. Her haemoglobin dropped to 7.3 g/dl and the platelet count dropped to 47,000/µl. The blood picture revealed evidence of microangiopathic haemolytic anaemia (MAHA), together with raised serum Lactate dehydrogenase levels of 1240 µ/l and normal coagulation profile. Other investigations on day 6 are summarized in Table 1. As the patient was not stable enough to undergo Magnetic resonance imaging (MRI) of brain, a non contrast CT (NCCT) brain was done on the same day and it did not reveal any mass effects suggestive of cerebral abscesses. Based on these findings, a presumptive diagnosis of thrombotic thrombocytopenic purpura (TTP) was made and the patient was commenced on plasmapheresis. ADAMTS 13 (a disintegrin and metalloprotease with thrombospondin type I, domain 13) assay was not done as it was not immediately available in our low resource setting. Following two cycles of plasmapheresis, patient’s neurological and haematological parameters improved, but the fever was still persisting with worsening chest x ray findings and renal abscess size. Contrast enhanced CT (CECT) abdomen revealed bilateral pyelonephritis with right upper pole renal abscess of 1.6 cm in diameter, early abscess formation in left lower pole and was negative for abscesses elsewhere or lymphadenopathy (Fig. 3).

Since there was a suggestive occupational history with multi-organ involvement and abscess formation, with poor response to initial broad spectrum antibiotics, melioidosis was suspected and antibody level was requested. Melioidosis antibody, which was measured by indirect haemagglutination, was highly positive with a titer of 1:2560. Based on the suggestive clinical features, exposure history and highly positive antibody titer, the patient was diagnosed to have melioidosis with severe pneumonia and bilateral pyelonephritis with renal abscess formation, complicated with TTP and severe sepsis.

**Fig. 4:**
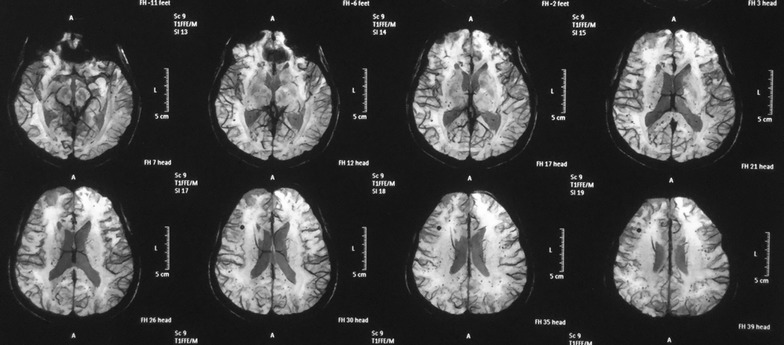
Magnetic resonance imaging of brain. Showing extensive minute haemorrhagic foci throughout the cerebrum

Intravenous meropenem 2 g 6 hourly and oral co-trimoxazole 1920 mg 12 hourly was started on day 7, following which, the patient demonstrated a dramatic clinical response, with fever settling within 48 h and gradual radiographic resolution. She was extubated on day 17. MRI of brain which was done following extubation was negative for cerebral abscesses but revealed extensive cerebral petechial haemorrhages with white matter oedema (Fig. 4). This was likely to be due to a consequence of co-existing TTP and thrombotic microangiopathy.

After continuing meropenem for 21 days, the patient was discharged on co-trimoxazole (1920 mg 12 hourly) to be taken up to 3 months. She was advised to refrain from breast feeding until the treatment is complete. The recovery was uncomplicated and she showed remarkable improvement in the follow up visits. After 4 weeks of treatment, melioidosis antibody titer reduced to 1:320, and the antibody was absent in the baby.

## Discussion and conclusions

*Burkholderia pseudomallei* is inherently resistant to initial empiric antibiotic regimens used to treat pneumonia or sepsis. Thus, melioidosis is a disease which could bring potentially fatal outcomes if not diagnosed early. Our patient is a previously healthy, young female who was in her early postpartum period, who presented with features of a severe pneumonia and sepsis. Her inadequate response to empiric antimicrobial therapy, and development of pyelonephritis with renal abscesses urged us to consider an alternative diagnosis. As her exposure history to surface water in paddy fields and highly suggestive clinical picture was compatible with a diagnosis of melioidosis, we proceeded with testing of antibody titer despite non yielding cultures. The antibody titer is measured via indirect haemagglutination. In Sri lanka *B. pseudomallei* is confirmed via Analytical profile index 20NE, with or without preliminary identification via PCR based methods. Although a positive culture is required for the diagnosis, it is well known that this could be misidentified as another organism in laboratories which are unfamiliar with *B. pseudomallei*. Therefore, the sensitivity of culture is low. As culture in the standard media will not identify the exact organism, it should be cultured in a specialized culture medium (Ashdown agar) [4, 5, 8]. Although her BALF culture isolated *S. maltophilia,* it was not considered significant in the current context as it is a gram negative bacterium which is known to colonize the respiratory tract of ventilated patients, rather than leading to a severe pneumonia of this nature [9].

Usual chest x-ray findings in acute melioidosis are described as diffuse or patchy lobar or multi lobar consolidation and pleural effusion. It is well documented that initial chest x-ray may only show very minimal infiltrates than what is expected in a severe pneumonia of this nature, which was observed in our patient’s initial chest radiograph as well [2]. Although genitourinary involvement and soft tissue abscesses are well documented manifestations of melioidosis, renal abscess formation had not been frequently reported with the acute disease, which signifies another importance of this case. Urine culture negativity in our patient may have been contributed by treatment with antibiotics prior to collection of the culture, and lack suspicion about the pathogen at this point.

Treatment of melioidosis consists of two phases; intensive phase and oral eradication therapy phase. The antibiotics recommended in the intensive phase are intravenous meropenem or ceftazidime. Although there is no proven benefit, it is practiced in some centers to add trimethoprim–sulfamethoxazole (TMP–SMX) to the intensive phase for the patients with soft tissue melioidosis [10, 11]. As our patient had severe pneumonia as well as multiple renal abscesses, we opted to add weight adjusted dose of TMP–SMX to the high dose meropenem in the initial intensive phase. The usual recommended duration for the initial intensive phase is 10–14 days. Four to eight weeks of therapy is recommended in critically ill patients with severe pulmonary disease or deep seated abscesses [10]. Therefore, in our patient, initial intensive therapy was continued for 21 days in keeping with the above recommendations. The recommended drugs to be used for oral eradication are TMP–SMX, doxycycline or amoxicillin–clavulanate, all to be continued for 3 months [10]. In our patient, we continued TMP–SMX up to 3 months for the oral eradication.

Patient was also diagnosed to have co-existing TTP presumptively based on the new onset seizures, thrombocytopenia and microangiopathic hemolytic anaemia. Subsequent MRI brain findings of cerebral petechial hemorrhages and prompt response to plasmapheresis were supportive for the above diagnosis.

Transmission via breast feeding has been described with *B. pseudomallei* mastitis [12] and there are a very few case reports on trans-placental transmission as well [3, 13]. However, in our case, antibody was not detected in the child.

This case was challenging as it was an atypical presentation of melioidosis during postpartum in a previously healthy patient leading to a diagnostic, as well as management dilemma. Although she did not have any of the well documented risk factors for the acute disease, the relative immunosuppressive status during pregnancy could have been contributory. The only clue that we had for the correct diagnosis was her history of occupational exposure. Despite the fact that this was a complicated and a challenging case for diagnosis as well as for management in a low resource setting as ours, the timely diagnosis and initiation of correct antibiotics without a delay helped to achieve successful results. Commonly used culture media in Sri Lanka such as Blood and McConkey agar do not give a specific colony morphology characteristic for *B. pseudomallei.* Therefore, detailed clinical history, higher degree of suspicion, and better communication between the treating physician and laboratory physician would lead to better diagnostic yield.

This case demonstrates an atypical presentation of acute melioidosis giving rise to pneumonia and multiple renal abscesses in a post partum woman. This highlights the importance of having a high clinical suspicion to avoid diagnostic delays of melioidosis even in patients without the classic risk factors. Even if the initial cultures are not suggestive, a very high antibody titer, together with the suggestive clinical picture and an exposure history can support the diagnosis. This case emphasizes the need to look into the effect of pregnancy and postpartum as added risk factors for the development of acute melioidosis. It is important to test the antibody level of the baby as there is a possible risk of trans-placental transmission and transmission via breast milk.

## Abbreviations

PCR: polymerase chain reaction
MRC: Medical Research Council
BMI: body mass index
ECG: electrocardiogram
WBC: white blood cell
CRP: C reactive protein
ESR: erythrocyte sedimentation rate
CTPA: Computed tomography pulmonary angiogram
HRCT: High-resolution computed tomography
BALF: Broncho-alveolar lavage fluid
MAHA: microangiopathic haemolytic anaemia
MRI: magnetic resonance imaging
NCCT: Non contrast computed tomography
TTP: thrombotic thrombocytopenic purpura
CECT: Contrast enhanced computed tomography
TMP-SMX: trimethoprim/sulfamethoxazole
